# Dexterous Manipulation Based on Object Recognition and Accurate Pose Estimation Using RGB-D Data

**DOI:** 10.3390/s24216823

**Published:** 2024-10-24

**Authors:** Udaka A. Manawadu, Naruse Keitaro

**Affiliations:** Graduate School of Computer Science and Engineering, University of Aizu, Aizu-Wakamatsu, Fukushima 965-0006, Japan

**Keywords:** 3D object recognition, 3D pose estimation, dexterous manipulation, point cloud

## Abstract

This study presents an integrated system for object recognition, six-degrees-of-freedom pose estimation, and dexterous manipulation using a JACO robotic arm with an Intel RealSense D435 camera. This system is designed to automate the manipulation of industrial valves by capturing point clouds (PCs) from multiple perspectives to improve the accuracy of pose estimation. The object recognition module includes scene segmentation, geometric primitives recognition, model recognition, and a color-based clustering and integration approach enhanced by a dynamic cluster merging algorithm. Pose estimation is achieved using the random sample consensus algorithm, which predicts position and orientation. The system was tested within a 60° field of view, which extended in all directions in front of the object. The experimental results show that the system performs reliably within acceptable error thresholds for both position and orientation when the objects are within a ±15° range of the camera’s direct view. However, errors increased with more extreme object orientations and distances, particularly when estimating the orientation of ball valves. A zone-based dexterous manipulation strategy was developed to overcome these challenges, where the system adjusts the camera position for optimal conditions. This approach mitigates larger errors in difficult scenarios, enhancing overall system reliability. The key contributions of this research include a novel method for improving object recognition and pose estimation, a technique for increasing the accuracy of pose estimation, and the development of a robot motion model for dexterous manipulation in industrial settings.

## 1. Introduction

In recent years, research into object recognition and pose estimation systems using three-dimensional (3D) data has grown in popularity and the costs of RGB-D camera sensors have been reduced significantly [1,2,3]. The introduction of Microsoft Kinect paved the way for a large number of RGB-D sensors that are now widely available, including Intel RealSense, Asus Xtion Pro, Creative Senz3D, and Structure Sensor. RGB-D cameras offer several advantages over other range sensors, such as light detection and ranging (LiDAR), including cost-effectiveness, low power consumption, higher frame rates, and high-resolution color data [4,5]. Especially when it comes to point clouds (PCs), the additional color information given by RGB-D cameras could increase their accuracy in scenarios that require color and depth data, such as object recognition. The availability of low-cost range-sensing technologies has contributed significantly to the improvements in research using RGB-D cameras. RGB-D images are typically captured using two primary methods; passive and active sensing [5]. Passive sensing involves capturing two images of the same scene at once using two cameras placed next to each other, while active sensing uses an infrared sensor to calculate the time it takes for the signal to bounce back [5]. Intel RealSense cameras combine both passive and active sensing while capturing images, as they feature two infrared cameras and an infrared projector to mitigate the noise caused by infrared radiation [5].

Six-degrees-of-freedom (6-DOF) pose estimation refers to the process of determining the position and orientation of an object in 3D space for a reference frame, as represented by the three dimensions of X, Y, and Z and the three angles of roll, pitch, and yaw, respectively. Recent developments in 6-DOF pose estimation have significantly enhanced its accuracy and robustness. Researchers have developed advanced models and learning-based methods for 6-DOF object recognition and pose estimation [6], which have led to increasingly precise and reliable manipulation tasks in robotic systems. Multiview data acquisition has played a crucial role in improving the accuracy of pose estimation. Additionally, the integration of advanced sensor technologies has allowed for the better handling of complex environments. Even with these improvements, challenges such as handling occlusions, varying lighting conditions, and the need for real-time processing still exist. Addressing these challenges is essential for advancing the field and enabling broader applications in industrial automation.

**Figure 1:**
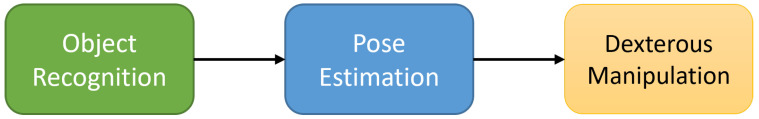
A simple schematic diagram of integrated systems used in previous research.

Model-based methods are mathematical and visual approaches to addressing problems related to designing complex controls. In the context of 3D object recognition and pose estimation, model-based methods are often preferred for their simplicity and the fact that they do not necessarily rely on a dataset, since creating a sizeable 3D dataset can be challenging. Unlike learning-based methods, which use machine learning algorithms to train models on large datasets of 3D object models, model-based methods can operate effectively without extensive data. This is particularly advantageous for industrial settings where collecting and labeling a large amount of 3D data can be impractical and time-consuming. Moreover, learning-based methods that rely on publicly available datasets often face limitations in their scalability and adaptability to specific industrial environments [7,8,9,10]. Integrating 3D object recognition, 3D pose estimation, and dexterous manipulation is essential for factory automation systems, particularly those involving robotic hands. As illustrated in Figure 1, integration systems typically perform 3D object recognition as the first step to identify the target 3D objects, followed by 3D pose estimation to determine the objects’ locations, and finally manipulate a robot hand to operate the target.

A complex 3D environment can be broken down into geometric primitives to facilitate data analysis and comprehension [11]. In the domain of 3D object recognition using model-based methods, segmenting raw PC files serves as a critical initial step in scene segmentation. Geometric primitives, such as planes, spheres, and cylinders, provide a simplified representation of complex structures, making it easier to analyze and identify objects within a scene. This simplification aids in reducing computational complexity and improving the robustness of the recognition process. Commonly employed scene segmentation methods for this purpose in PC include region growing, graph-based techniques, and clustering [12]. Moreover, some researchers have used additional feature extraction or filtering techniques to further refine the accuracy of object recognition [13]. Geometric primitives allow model-based methods to align segmented portions of the PCs more accurately with predefined models, thereby improving the precision and effectiveness of object recognition and pose estimation.

Pose estimation is the process of determining the 6-DOF pose of an object efficiently using 3D data. Advanced techniques apply matching or voting strategies with local or global features to generate preliminary hypotheses and refine the 6-DOF pose accordingly when 3D models of the object are available [14]. Examples of currently available model-based 3D pose estimation methods include random sample consensus (RANSAC), Hough voting, iterative closest point (ICP), and the centroid connection method. However, the limited training datasets make it challenging to implement 3D pose estimation for unknown objects [14]. Recent research findings have shown that employing multiview data acquisition techniques significantly enhances the accuracy of pose estimation compared to relying on single-view approaches [15,16,17]. By obtaining multiple perspectives of the objects or scenes, multiview methods provide a more comprehensive and nuanced understanding of their spatial configurations, resulting in more precise and reliable pose estimations.

Systems that integrate object recognition and pose estimation often do not incorporate dexterous manipulation, which refers to the ability to control hands skillfully and precisely for object manipulation and is a desirable feature for such systems [14]. Most commercially available robot arms are capable of dexterous manipulation. Previous studies have mainly focused on object picking robots and have employed simple inverse kinematics (IK) techniques for dexterous manipulation [1,14]. In the use case that we consider in this work, however, precise robot hand manipulation is necessary. Our literature review indicates that improving robot hand manipulation can also enhance object recognition and pose estimation performance. It is essential to integrate object recognition, pose estimation, and dexterous manipulation to create seamless and efficient workflows in robotic systems, which allows them to execute complex tasks with high precision and reliability. This comprehensive approach not only enhances each component but also boosts the overall performance of the system in industrial automation environments.

In this study, we propose a novel integrated system for object recognition, pose estimation, and dexterous manipulation that employs an RGB-D camera mounted on a 6-DOF robot arm, which captures PCs from multiple perspectives to enhance the accuracy of pose estimation to make the dexterous manipulation of target objects easier. This multiview approach allows for a more comprehensive understanding of the objects’ spatial configuration by overcoming challenges such as occlusions and varying lighting conditions. Specifically, this approach is employed when the object is located at one side of the camera. This research specifically focuses on automating the opening and closing of diverse types of valves using the robot arm, which requires precise control and flexibility for various valve designs. By integrating these three key functionalities into a single system, we aim to demonstrate significant improvements in the efficiency and reliability of robotic manipulation tasks in industrial settings. The key contributions of this research are as follows:

- The development of a novel, more accurate method for object recognition, pose estimation, and dexterous manipulation using a model-based approach.
- The introduction of a new technique to increase the accuracy of pose estimation by moving the camera to different positions and collecting more precise RGB-D data than existing techniques.
- The creation of a conventional robot motion model for dexterous manipulation to efficiently open and close various valves.

The remainder of this paper is organized as follows. Section 2 presents an in-depth analysis of the previous works and highlights the necessity of this research. Section 3 introduces the novel system architecture of the proposed object recognition, pose estimation, and dexterous manipulation algorithm. Section 4 and Section 5 present technical details about the proposed object recognition and pose estimation modules, respectively. Section 6 explains the experimental setup and data collection methods in this research. The experimental results and an evaluation of the effectiveness and accuracy of the proposed system are presented in Section 7. Section 8 and Section 9 conclude with this research’s contributions and suggest potential future works.

## 2. Literature Review

In this literature review, we present the previous research on 3D geometric modeling, simultaneous 3D object recognition and pose estimation, and dexterous manipulation within the context of 3D object recognition and pose estimation, along with their respective limitations.

### 2.1. 3D Geometric Modeling

The process of converting a complex PC environment into 3D geometric primitives involves several data preprocessing steps, including noise removal, downsampling, and surface normal estimation [18,19]. Geometric primitives consist of basic shapes, such as circles, planes, cubes, spheres, cylinders, cones, ellipsoids, and prisms, which are combined to form more complex solid objects [20]. Techniques like region growing, RANSAC, and clustering are then used to extract geometric primitives by grouping points into shapes, such as planes, lines, or cylinders [11]. These extracted geometric primitives serve as foundational elements for object extraction and reconstruction, among other application-specific processing tasks, which facilitate deeper analyses and understanding of the 3D environment derived from the PC data.

Segmenting the captured scene and identifying segments as simple geometrical objects verifies the object recognition process. Detecting and filtering simple objects within the PC data, such as handles, circles, or rectangles, can significantly enhance the accuracy of object recognition [21]. By isolating these basic shapes from a given scene, the presence and position of more complex structures can be confirmed. This approach is particularly relevant to our research, as it makes it easy to precisely identify and manipulate industrial components, such as valves. By maximizing the advantages of 3D geometric modeling, our system can achieve increased accuracy in pose estimation and manipulation tasks, ultimately improving the efficiency and reliability of robotic automation in industrial settings.

### 2.2. Simultaneous 3D Object Recognition and Pose Estimation

Tsai et al. proposed a system for 3D object recognition and pose estimation that effectively identifies and estimates the pose of an object of interest (OOI), even when it is occluded or the camera is rotated [1]. In this research, the 3D object recognition module consists of four units: scene segmentation, feature description, descriptor matching, and Hough voting. Scene segmentation removes background points in the colored PCs obtained from the RGB-D camera. The feature description unit constructs feature descriptors of all foreground objects in the scene. The descriptor matching and Hough voting units then identify the OOI and provide its initial pose information. The pose estimation scheme refines the initial pose of the OOI using the RANSAC algorithm and a hypothesis verification algorithm to achieve the best results for the pose estimation of objects. However, this research is limited to object-picking scenarios and does not address dexterous manipulations.

Guo et al. presented a new method for object recognition and 6-DOF pose estimation based on point pair features (PPF), which improves the entire pipeline of previous PPF-based methods. They introduced a novel center-targeted voting scheme that generates new points near the objects’ centers in the scene, which enhances the robustness and computational efficiency of the method [22]. However, this research heavily depends on the availability of public 3D PC datasets. Parisotto et al. proposed a method that enables robots to recognize objects and estimate their poses concurrently by sharing representations between these tasks [15]. This research is based on a deep object-agnostic entropy estimation model that predicts the most informative viewpoints for a given 3D object. By inputting these predicted views into a viewpoint network, the system can simultaneously predict the pose and category labels for the target object. Jantos et al. introduced pose estimation transformer (PoET), which is a novel transformer-based framework for accurately estimating the 6-DOF poses of objects in single-view RGB images without requiring additional depth maps or 3D models [23]. The PoET approach influences object detection and transformer networks to predict object translation and rotation simultaneously. By considering the entire image during estimation, PoET is better than the other RGB-based methods regarding its performance on the challenging YCB-V dataset.

However, these studies on simultaneous 3D object recognition and pose estimation are constrained by several limitations, including their dependence on instance segmentation accuracy, reliance on specific computer-aided design (CAD) models for testing, and difficulties in generalizing to new environments. Additionally, these methods face potential limitations in their evaluation metrics, sensitivity to noise and occlusions, and the impact of limited dataset diversity on performance. Despite these challenges, the integration of robust pose estimation techniques with dexterous manipulation is essential for improving robotic automation in industrial settings. Our research aims to address these limitations by developing a model-based approach that leverages multiview data acquisition to enhance the accuracy and reliability of pose estimation.

### 2.3. Dexterous Manipulation in 3D Object Recognition and Pose Estimation

Research on dexterous manipulation within the context of 3D object recognition and pose estimation is limited. Wen et al. introduced a framework for robust within-hand 6-DOF pose estimation using a consumer-level depth sensor based on the adaptive Yale Hand T42 [24]. Although this approach addressed the challenges associated with adaptive hands, it was mainly tested on a custom dataset created by the authors and lacked real-time testing. Sundermeyer et al. presented a method named “Contact-GraspNet”, which simplified the 6-DOF grasp estimation problem by transforming it into grasp contact point classifications and grasp rotation estimations [25]. This method achieved a higher grasp success rate in cluttered real-world scenes compared to previous approaches, which demonstrated its effectiveness in practical applications. Li et al. developed a context-aware anthropomorphic robotic hand called “Magic Hand”, which was designed for dexterous grasping. This robotic hand is equipped with sensors that provide feedback to facilitate the grasping of objects [26]. The integration of sensors allows for adaptive and precise manipulation, enhancing its practical use.

Hernando et al. conducted a study focused on improving the accuracy of hand pose estimation in physics-based tasks using a combination of reinforcement learning (RL) and imitation learning (IL) [27], which explored various scenarios such as door opening, in-hand manipulation, tool use, and object relocation for dexterous manipulation. Their hybrid RL+IL approach showed significant improvements in the accuracy of hand pose estimation. Their findings identified challenges for accurate 6-DOF pose estimation, especially when precise object segmentation is needed, showing the importance of testing estimation methods using real-world situations. Their research on dexterous manipulation and pose estimation is limited by its reliance on specific datasets and environments, a lack of real-world testing, and potential difficulties in generalizing to diverse scenarios.

### 2.4. Summarizing the Previous Works

Table 1 provides a summary of similar works carried out previously. The literature highlights the necessity for developing an integrated system capable of simultaneously performing object recognition, pose estimation, and dexterous manipulation to enhance efficiency in robotic tasks. Specifically, past studies have identified the lack of availability of 3D datasets when applying learning-based methods. Given this limitation, model-based approaches can be employed for object recognition and pose estimation in scenarios where public 3D datasets are unavailable. Studies have shown that obtaining data from multiple angles can significantly improve the accuracy of pose estimation. In our study, we used an Intel RealSense camera mounted on a JACO robot arm with six DOF to capture data from various angles. Since our focus is primarily on shutting multiple kinds of valves on or off, the camera is positioned at angles conducive to the increased accuracy of object pose estimation. Specifically, previous research states that the accuracy of pose estimation for objects is higher when the camera is positioned in front of them. This research involves moving the camera to optimal positions in front of the objects to enhance the accuracy of object recognition and pose estimation. By manipulating the objects accordingly, we aim to achieve higher precision and reliability in robotic automation tasks.

## 3. System Architecture

**Figure 2:**
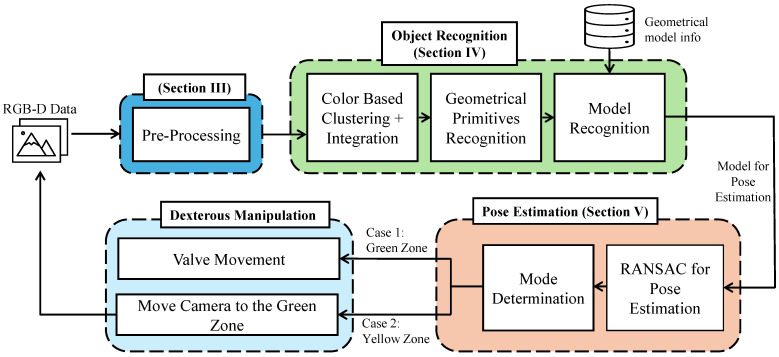
System architecture for the proposed system for object recognition, pose estimation, and dexterous manipulation. The methodology for preprocessing is described in detail in Section 3, object recognition is discussed in Section 5, pose estimation is covered in Section 5.1, and information about the yellow and green zones is provided in Section 5.2.

Figure 2 illustrates the simplified architecture of the proposed object recognition, pose estimation, and dexterous manipulation algorithm. The system contains three main modules: an object recognition module, a pose estimation module, and a dexterous manipulation module. The robotic system is implemented using the C++ language, the Point Cloud Library (PCL), the Eigen Library, and the Intel RealSense Software Development Kit (SDK) 2.0 [30,31,32]. In this research, we opted for well-established, widely used algorithms because they offer a balance between reliability and efficiency without imposing additional constraints on the system. These algorithms are known for their robustness and adaptability across a variety of environments, making them a natural fit for industrial applications such as object recognition and pose estimation. By leveraging these proven methods, we ensure that the system operates effectively in real-world scenarios without requiring complex modifications or specialized conditions.

Initially, color PC images captured from the RGB-D camera are preprocessed to enhance the quality and accuracy of the PC scene before being fed into the object recognition module, which consists of three submodules: color-based clustering and integration, geometrical primitives recognition, and model recognition. Its primary function is to identify the target object through a filtering process from the given scene. The pose estimation module is implemented to enhance the accuracy of the initial pose determined by the object detection module for the target object. In this research, the RANSAC algorithm was employed to refine the pose of the target object, specifically for the handles of both globe and ball valves. RANSAC was selected due to its well-known ability to handle noise and outliers effectively.

**Figure 3:**
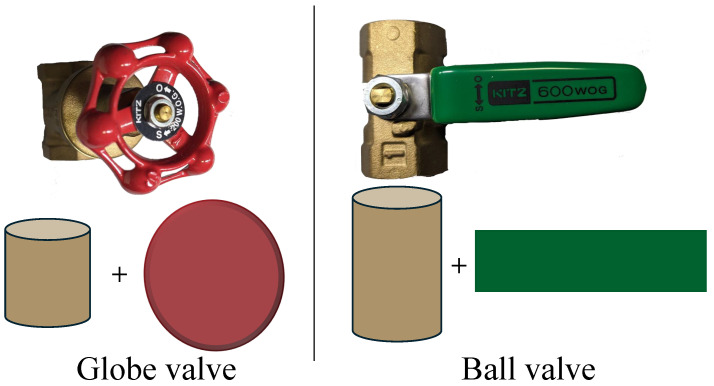
Geometric abstractions of valve types. The globe valve (**left**) is represented by a combination of a cylinder and a disc, and the ball valve (**right**) is depicted as a cylinder and a plane.

As illustrated in Figure 3, a valve comprises two main components: the handle and body. The primary objective of this research was to manipulate the valve handle accurately using a robot arm from different directions. Therefore, achieving precise positioning of the handle is crucial in this context. Previous studies have shown that using multiple viewpoints can enhance the accuracy of object recognition and pose estimation [15,16,17]. When the RGB-D camera is positioned directly in front of the target object, the accuracy of object detection and pose estimation is higher, enabling smoother valve movement, which was mostly achieved in previous bin picking studies [22,33,34]. If the RGB-D camera is situated to the side of the target object, however, the acquired pose estimation data from the RANSAC algorithm prompt the RGB-D camera to reposition itself directly in front of the object. This methodology in this research ensures the accurate acquisition of pose estimation data (as in Figure 6). In this use case scenario, the accuracy of the valve handle pose can be improved by capturing the valve from the front, which provides the highest amount of information about the position and orientation of the valve. Moving the camera to the front can be achieved through coordinated movement of the robot arm with an RGB-D camera, because the camera is attached to the robot arm.

The dexterous manipulation module is integrated with the output from the pose estimation module, which enables the robot arm to maneuver itself to the front of the target object to capture PCs more accurately than other methods. Additionally, it facilitates the execution of predetermined valve movements, such as opening or closing, based on the identified pose of the valve.

### Preprocessing Filters

In this study, an Intel RealSense D435 camera was used as the depth sensor [35]. The Intel RealSense D435, with its infrared projectors and sensors, allows for depth perception, making 3D mapping and object tracking much easier. To enhance the quality and accuracy of the captured depth data, postprocessing steps using Intel RealSense’s SDK 2.0 were implemented [32]. The SDK provides Smooth Alpha and Smooth Delta filters to control the level of smoothing applied to the depth data. Specifically, the Smooth Alpha filter is designed to reduce noise, while the Smooth Delta filter assists in smoothing out depth information. Additionally, a hole-filling filter from the SDK is used to address missing data points in the depth map. This filter is crucial for filling gaps or areas with incomplete depth information, resulting in a comprehensive and coherent representation of the 3D scene.

## 4. Object Recognition

This section introduces the three submodules of the object recognition module, namely scene segmentation & integration, geometrical primitives recognition, and model recognition.

### 4.1. Color-Based Clustering & Integration

Clustering the 3D PCs is necessary for extracting meaningful information and understanding the spatial relationships within the data, as they significantly enhance object detection by offering contextual information, semantic insights, and localization cues, which together boost the efficiency and accuracy of detection algorithms, especially when dealing with occlusions [36]. The color-based region-growing segmentation algorithm offers several advantages over other scene segmentation algorithms by obtaining color information from PCs, unlike traditional segmentation algorithms that rely only on geometric properties [36]. This process increases the comprehensiveness of scene analyses by incorporating geometric and color features for segmentation.

Due to the noise in real-time captured PC data, color-based segmentation might not obtain perfect clustering for the target objects in this research, namely valve handles and valve bodies. Hence, a dynamic cluster merging algorithm was introduced based on color and proximity to enhance the clustering accuracy of PC data. The algorithm is presented in Algorithm 1. First, clusters obtained from region-growing segmentation are filtered by color to create further recognition of the target object. Color filtering is performed by converting the color information of clustered PCs to the hue, saturation, value (HSV) color space and then calculating the average color values [37]. The choice of HSV color space for color-based segmentation is grounded in its alignment with human color perception and its decoupling of color intensity from color information, making it more robust to variations in lighting compared to RGB space [37]. This color space creates more consistent clustering by averaging color values within clusters to reduce the impact of noise in real-time-captured PC data. After that, the algorithm determines the centroid of each cluster for every color category and identifies the nearest cluster within the merged clusters based on the distance threshold. If a nearby cluster is found, the current cluster is merged with it; otherwise, the cluster is added to the merged clusters list. Additionally, if a cluster cannot be merged based on proximity, the algorithm identifies the largest subcluster using Euclidean cluster extraction, which is then added to the merged clusters. This approach can effectively group clusters based on color consistency and spatial proximity, thereby improving the overall segmentation accuracy of the PC data. The output gives a list of integrated clusters based on color and proximity, which is discussed in the geometrical primitives recognition subsection.
**Algorithm 1** Dynamic cluster merging based on color and proximity**Inputs:**  **C**: Vector of point indices representing clusters from region-growing segmentation  *d*_threshold_: Base threshold for centroid distance**Intermediate Data:****F**: Clusters filtered by color**M**: Clusters merged based on color and distance**Algorithm Steps:**1:Initialize **F** and **M** as empty lists2:Define color ranges for HSV values3:**for** each cluster **C**[*i*] in **C** **do**4:    hsv ← ConvertToHSV(**C**[*i*])5:    color_category ← ClassifyColor(hsv, color ranges)6:    **if** color_category is not null **then**7:        Add **C**[*i*] to **F** under color_category8:    **end if**9:**end for**10:**for** each color_category in **F** **do**11:    **for** each cluster **F**[*j*] in color_category **do**12:        centroid ← CalculateCentroid(**F**[*j*])13:        nearest_cluster ← FindNearestCluster(centroid, **M**, *d*_threshold_)14:        **if** nearest_cluster is not null **then**15:           Merge **F**[*j*] with nearest_cluster16:        **else**17:           Add **F**[*j*] to **M**18:           **E** ← getEuclideanClusterExtraction(**F**[*j*])19:           Add **E** to **M**20:        **end if**21:    **end for**22:**end for****Output:****M**: List of integrated clusters based on color and proximity

### 4.2. Geometrical Primitive Recognition

The RANSAC algorithm is used to identify primitive shapes within the segmented PC data. Widely known for its robustness to outliers, RANSAC is particularly specialized at fitting models to data with a significant proportion of noise [38]. For this development, this research uses RANSAC in PCL [30]. In this research, globe and ball valves are categorized as simple geometrical features (Figure 3). The globe valve can be simplified as a cylinder and a disc to represent the main body and the valve wheel, respectively. Similarly, the ball valve is reduced to a cylinder to represent its body, and the handle can be represented by a rectangular plane. Similarly, any kind of valve can be derived as geometrical primitives, and the RANSAC algorithm is used to identify them. RANSAC is particularly suited for this type of model fitting process due to its ability to fit simple geometrical shapes with minimal parameters while maintaining computational efficiency, which ensures that the algorithm not only identifies these primitives accurately but also does so in a manner that is computationally feasible for real-time or near real-time applications [39].

This robotic system is designed to identify more than one geometrical primitive within a single cluster. This multiplicity is essential, as real-world objects are often made up of several geometric forms. After applying the RANSAC algorithm, the geometrical primitives are further classified by their dimensions. The globe valve handle’s radius, the ball valve handle’s width, and the body cylinder’s parameters will be given to the system.

### 4.3. Model Recognition

The model recognizes and distinguishes whether the processed PC with color and geometrical information represents a ball or globe valve. This identification is essential for determining the initial pose estimation for each recognized cluster because pose estimation for different models uses different methods. To achieve this identification, we use a predefined model description in an external JavaScript Object Notation (JSON) file containing model data. JSON’s human-readable format and efficacy in representing complex data structures in a text-based format motivate the choice of JSON files [40]. This JSON file contains the model’s name, color, shape, dimensions, and the translation and rotation parameters of each model.

**Figure 4:**
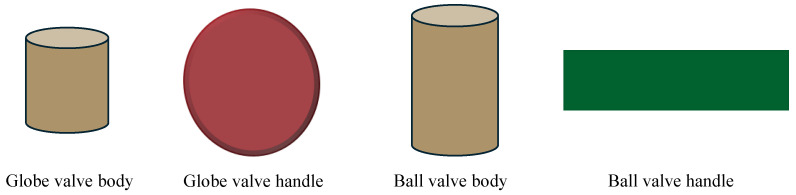
Output of the object recognition module.

The output from the object recognition module includes a model, which can represent a body part, a handle, or both a body part and a handle. If it recognizes both the body part and the handle, each identified object is assigned specific translation and rotation values, which are predefined in the JSON file. This approach creates accurate positioning and orientation of each model in preparation for the subsequent robotic manipulation tasks. The process by which this information feeds into pose estimation is illustrated in Figure 4.

## 5. Pose Estimation

The outputs from the object recognition module are then passed to the pose estimation module, which has the task of accurately determining the position and orientation of the objects identified by the object recognition process and sending them to the manipulation process. The position identified from the pose estimation module is usually the center of the handle. The orientation of the object is calculated as Orientation X (O_x_), Orientation Y (O_y_), and Orientation Z (O_z_). From the O_z_, which is often called the "Forward” vector, it is easy to gain an idea about the positioning of the valve.

### 5.1. RANSAC for Pose Estimation

The RANSAC algorithm was used for pose estimation in this research. The geometrical data from the object recognition module was applied across different RANSAC algorithms to determine the pose. The RANSAC algorithm was used as shown:

- Globe valve handle: RANSAC Circle
- Ball valve handle: RANSAC Plane

#### 5.1.1. RANSAC for Circle

This research implemented a RANSAC-based algorithm to estimate the pose of circular features by fitting a circle to a subset of 3D points from a PC and iteratively refining the model to identify the best-fit parameters. Below, we outline the key equations and steps used in the pose estimation process. The process starts by selecting three points $\left(p_{1},p_{2},p_{3}\right)$ from the PC. The normal vector n to the plane defined by these points is calculated using the cross product,

(1) $$n=\frac{\left(p_{2}-p_{1}\right)\times \left(p_{3}-p_{1}\right)}{∥\left(p_{2}-p_{1}\right)\times \left(p_{3}-p_{1}\right)∥}.$$

The center c of the circle is determined by solving a system of linear equations derived from the plane normal and the vectors connecting the points,

(2) $$c=A^{-1}B,$$

where *A* is the coefficient matrix and *B* is the vector constructed from the dot products of the connecting vectors. Specifically, *A* is defined as

(3) $$A=\left(\begin{array}{ccc} n_{x} & {\left(p_{2}-p_{1}\right)}_{x} & {\left(p_{3}-p_{1}\right)}_{x}\\ n_{y} & {\left(p_{2}-p_{1}\right)}_{y} & {\left(p_{3}-p_{1}\right)}_{y}\\ n_{z} & {\left(p_{2}-p_{1}\right)}_{z} & {\left(p_{3}-p_{1}\right)}_{z} \end{array}\right),$$

where $n_{x}$, $n_{y}$, and $n_{z}$ are the components of the normal vector n, and the components ${\left(p_{2}-p_{1}\right)}_{x}$, ${\left(p_{2}-p_{1}\right)}_{y}$, ${\left(p_{2}-p_{1}\right)}_{z}$, ${\left(p_{3}-p_{1}\right)}_{x}$, ${\left(p_{3}-p_{1}\right)}_{y}$, and ${\left(p_{3}-p_{1}\right)}_{z}$ are derived from the vectors connecting the points. The vector *B* is defined as

(4) $$B=\left(\begin{array}{c} n\cdot p_{1}\\ \frac{\left(p_{2}-p_{1}\right)\cdot \left(p_{1}+p_{2}\right)}{2}\\ \frac{\left(p_{3}-p_{1}\right)\cdot \left(p_{1}+p_{3}\right)}{2} \end{array}\right).$$

The radius *r* of the circle is then calculated as the Euclidean distance between the circle center c and one of the points, $p_{1}$,

(5) $$r=∥c-p_{1}∥.$$

O_z_ can be calculated as

(6) $$O_{z}=\frac{n}{∥n0∥}$$

**Figure 5:**
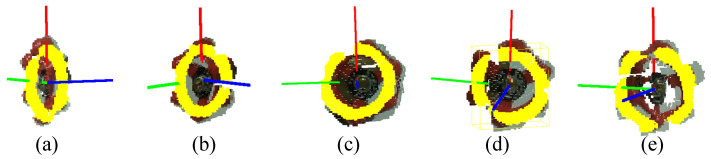
Position and orientation prediction using a RANSAC circle. Yellow points are inliers that are used for the RANSAC algorithm. In (**a**–**e**), images are depicted at a 30-degree interval on the yaw axis. (**a**) Pitch 0, yaw -60; (**b**) pitch 0, yaw -30; (**c**) pitch 0, yaw 0; (**d**) pitch 0, yaw 30; and (**e**) pitch 0, yaw 60.

The inlier points are evaluated based on their proximity to the circle model. For a given point *p*, an evaluation function f(p) is used to determine its fit to the model. Points satisfying the tolerance criteria are considered as inliers. Finally, the pose of the detected handle is computed using the normal vector n, the center c, and the reference points of the handle. This orientation is used to define the handle’s alignment in space, which is used for accurate robotic manipulation. Figure 5 shows the predictions of the position and orientation of the globe valve at different angles, where the center of the valve is considered as the position that will be predicted. In Figure 5, the start of the orientation is considered as the predicted position, and the red vector is O_x_, the green vector is O_y_, and the blue vector is O_z_).

#### 5.1.2. RANSAC for Plane

In this research, we implemented a RANSAC-based algorithm to estimate the pose of planar surfaces, particularly focusing on predicting the position and orientation of a ball valve. The algorithm identifies a plane within the 3D PC data and computes its normal vector, which is critical for estimating the valve’s pose. The aim is to identify the center of the handle, O_z_, which is the normal vector, and O_x_, which is the longest axis of the handle. Identifying the longest axis of the handle is important for accurately manipulating the ball valve from the robot’s hand. The process begins with the application of RANSAC to segment the planar surface from the input PC. The algorithm fits a plane model to the points using the following equation:

(7) ax+by+cz+d=0,

where *a*, *b*, and *c* are the coefficients representing the normal vector to the plane, and *d* is the distance of the plane from the origin. The RANSAC algorithm iteratively selects subsets of points and fits the plane model, identifying the inliers that lie within a predefined distance threshold from the model. The coefficients of the best-fitting plane are then extracted.

The normal vector n=(a,b,c) to the plane is normalized to obtain the orientation of the Z-axis ($O_{z}$) of the valve’s pose,

(8) $$O_{z}=\frac{n}{∥n∥}.$$

Next, the PC is centered by calculating its centroid c, which is given by

(9) $$c=\frac{1}{N}\sum_{i=1}^{N}p_{i},$$

where *N* is the number of points in the PC and $p_{i}$ represents each point. This centroid is used as a reference point for further calculations. The centroid c is defined as the best estimate for the position of the ball valve. Singular value decomposition (SVD) is performed on the centered PC to determine the orientation of the plane. The SVD decomposes the centered points into their principal components, yielding the direction vectors corresponding to the maximum variance in the data. The first principal component, $V_{1}$, represents the O_x_ of the valve’s pose,

(10) $$O_{x}=V_{1}.$$

O_y_ is computed as the cross product of the O_x_ and O_z_,

(11) $$O_{y}=O_{z}\times O_{x}.$$

### 5.2. Mode Determination for the Green and Yellow Zones

**Figure 6:**
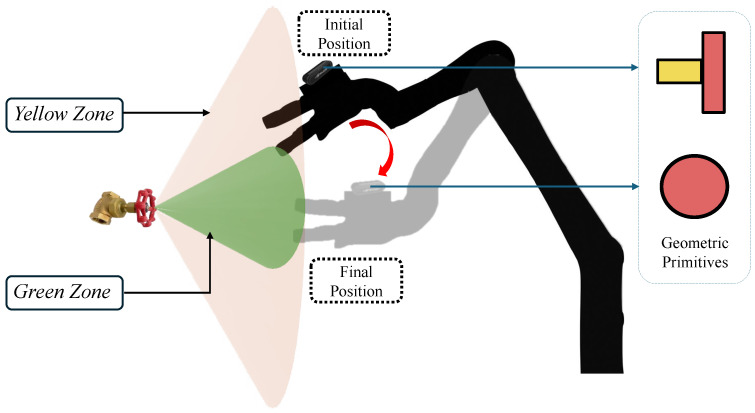
The “yellow” and “green” zones in the testing environment are shown in a schematic diagram of transitioning from the initial position in the yellow zone to the green zone’s final position. The range for the green zone is tested in this research.

To facilitate this pose estimation, we introduce two terminologies: namely, the “green zone” and the “yellow zone” (Figure 6). The green zone can be defined as a range with the minimum position and orientation error relative to the target object, while the yellow zone is defined as having position and orientation errors higher than the threshold values given for each of the valves. The threshold values are based on the geometrical shapes of the valve handles. These specific angle ranges were selected based on the geometric considerations of the valve and the field of view of the camera attached to the robot arm. The range for the green zone ensures that the camera is almost directly facing the valve, providing the most accurate data for pose estimation. The range for the yellow zone allows views where the camera is angled to the object, which might make pose estimation less reliable. The calculation for the range of the green and yellow zones is presented in the Results and Conclusions sections. The optimum robot arm angle (i.e., camera angle) in relation to the object and position is calculated based on in-depth experimental results. In the example shown in Figure 6, when considering the globe valve, the yellow zone fails to accurately recognize the valve handle (red line). In the green zone (depicted by a red disk in Figure 6, lower), however, the handle can be identified accurately to enable a precise estimation of its pose.

### 5.3. Dexterous Manipulation for the Globe and Ball Valves

The dexterous manipulation task consists of two parts. The first part, performed in the green zone, involves executing the valve movement, while the second part, performed in the yellow zone, involves moving the camera into the green zone. The valve movement differs for each type of valve. As shown in Figure 3, the valve part of the ball valve is recognized as a disk, and the valve part of the globe valve is recognized as a plane. The steps for dexterous manipulation are as follows:

(1) Check whether the robot arm’s current position is in the green or yellow zone. If it is in the yellow zone, move the robot arm to the green zone, then perform object recognition and pose estimation again.
(2) Move the robot arm to the standby position.
(3) Manipulate the valve.

**Figure 7:**
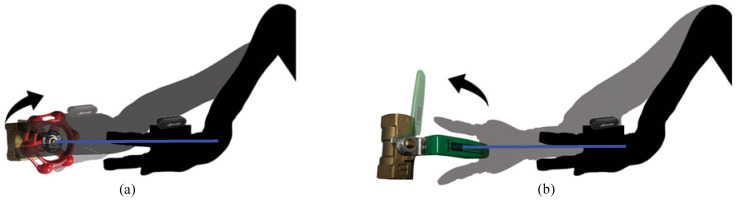
(**a**) Manipulation of the globe valve. (**b**) Manipulation of ball valve.

For dexterous manipulation, we feed a point on the valve, and the normal vector is calculated using RANSAC into the JACO arm SDK. A standby position is defined as 20 cm in front of the target object to ensure that the robot arm is correctly positioned before manipulating the valve. We selected the center point of the recognized object (the valve handle) as the target point for manipulation. The valve manipulation task is performed using inverse kinematics. Figure 7 illustrates how each valve is manipulated in this research. The blue line represents the normal vector estimated by the pose estimation module.

## 6. Experimental Setup

The proposed object recognition, pose estimation, and dexterous manipulation system was implemented with PCL in the C++ language [30] based on the Ubuntu 22.04 platform with Intel^®^ Core(TM) i7-12700 with 2.1 GHz and 32 GB system memory. An Intel RealSense D435 RGB-D camera was used to capture the 3D structure of the environment and objects. This camera has a minimum depth distance (Min-Z) of 28 cm at its maximum resolution, making it suitable for close-range tasks such as valve manipulation in industrial settings. A KINOVA^®^ JACO Gen2 6-DOF robot arm was used in this research. As shown in Figure 6, the Intel RealSense D435 camera was mounted on top of the robotic arm, which allowed the camera to have an optimal field of view for capturing the workspace, ensuring that the system could accurately identify and estimate the pose of objects in real-time as the arm approached and interacted with them. The experiment was carried out in a room under controlled lighting conditions to minimize shadows, glare, and other lighting-related artifacts that could interfere with the 3D perception system. The testing area was calibrated to establish the static position and orientation for the object and robotic arm base.

### Pitch and Yaw Terminology

**Figure 8:**
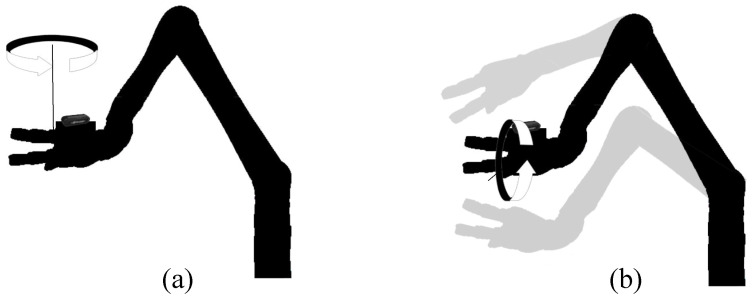
Depiction of pitch and yaw terminology: (**a**) yaw and (**b**) pitch axis changes.

The area of testing was considered as a 60° field of view in all directions in front of the object. To better represent our results, we used pitch and yaw terminology to represent the position of the robot arm. Figure 8 illustrates the pitch and yaw usage in this research, where Figure 8a illustrates pitch 0; yaw 0 positions are considered as being directly in front of the object and moving around the yaw axis (left-right), while Figure 8b illustrates the changes around the pitch axis. The shadows are some examples for pitch 15, yaw 0 (up), and pitch −15, yaw 0 (down). The JACO arm is inaccessible at certain yaw and pitch angles. In this research, we used scattered data interpolation to estimate data for positions that the JACO arm cannot physically reach [41]. The interpolated data are highlighted in bold and italics in tables presented in the Results and Discussion section.

## 7. Results and Discussion

### 7.1. Object Recognition Results

**Figure 9:**
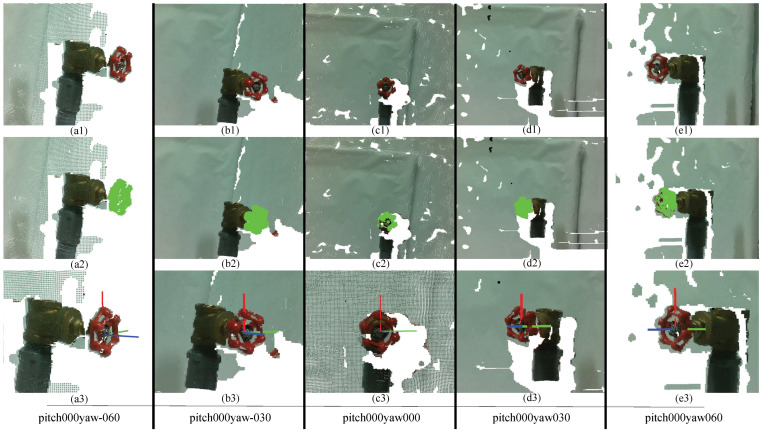
Experimental results of the 3D object recognition (sub-figures a2–e2) and pose estimation (sub-figures a3–e3) of a globe valve at different yaw angles in the pitch 000 axis.

**Figure 10:**
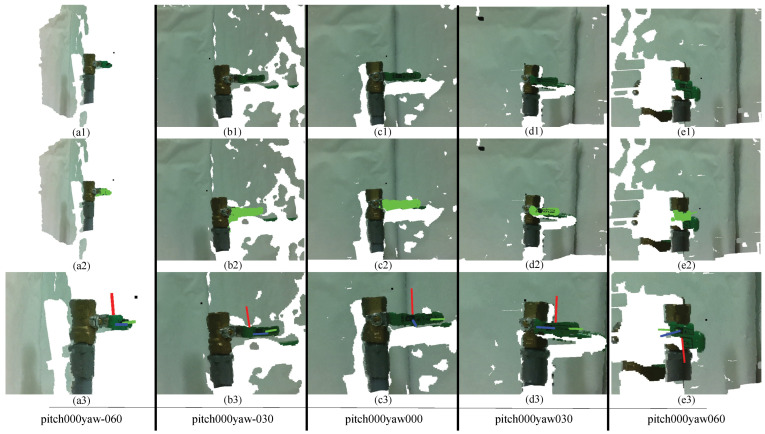
Experimental results of the 3D object recognition (sub-figures a2–e2) and pose estimation (sub-figures a3–e3) of a ball valve at different yaw angles in the pitch 000 axis.

The task of the object recognition module was to identify target objects, geometric primitive information, and model information for the pose estimation module. The object recognition module was tested in different scenarios where the camera was positioned at various angles in relation to the object, increasing the difficulty of accurate recognition. Figure 9(a2–e2) and Figure 10(a2–e2) show the 3D object recognition results using the proposed algorithm. Light green data points indicate the recognized object as determined by the object recognition module.

From these results, it is clear that the proposed object recognition system can successfully recognize the given objects under the current experimental settings. In extreme cases, however, such as when the pitch or yaw angle exceeds 60°, when objects are more than 70 cm away from the camera, or in situations like Figure 10a,e (pitch000yaw-060 and pitch000yaw060), where the handle of the ball valve is not clearly visible to the camera, the algorithm struggles to identify objects.

### 7.2. Pose Estimation Results

Different scenarios were considered for each of the valves to estimate the pose. Globe valves are semisymmetric, which means that independently changing either the pitch or yaw axes is sufficient for testing. In this research, we varied only the yaw axis of the robot arm’s position when testing the globe valve. In contrast, the ball valve is not semisymmetric, so both the pitch and yaw axes were varied for a comprehensive evaluation. Both position and orientation errors were considered in each scenario. Each testing scenario was repeated ten times, and the mean error values of position and orientation were recorded. Position and orientation accuracy were considered as the evaluation matrices of the pose estimation. The position error was measured in meters and varied depending on both the angle of the object relative to the camera and the distance between the object and the camera. The threshold for acceptable position error was set at 1.5 cm (0.015 m), and orientation error was set at 10° in this research.

The position error of valves can be calculated as

(12) $$Error_{pos}=∥C_{true}-c_{predicted}∥,$$

where $C_{true}$ is the target position of the object that was manually calibrated in the testing environment and $c_{predicted}$ is the target given by the pose estimation module that is given in Equations (2) and (9). The orientation error of the object was calculated as

(13) $$Error_{ori}=∥A_{true}-a_{predicted}∥,$$

where $A_{true}$ is the orientation of the target object that was manually calibrated in the testing environment, which is considered as the angle of the camera in relation to the object. $a_{predicted}$ is calculated according to the normal vectors given in Equations (6) and (8) and by adjusting the normal vector to the $A_{true}$.

#### 7.2.1. Pose Estimation Results for Globe Valve

**Figure 11:**
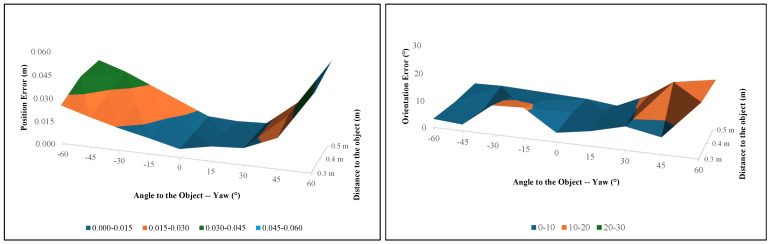
Surface plot for Table 2 and Table 3 for the globe valve. The left plot represents position errors, while the right plot represents orientation errors at different yaw angles and distances from the object. A blue-colored surface in the plot indicates the accepted threshold range.

Figure 9(a3–e3) and Figure 10(a3–e3) show the 3D pose estimation results for the globe value using the proposed algorithm. Table 2 and Table 3 and Figure 11 show how the position error changes from the different yaw angles and different distances to the globe valve. Table 2, Table 3 and Table 4 present the summarized pose estimation results. The bold and italic values inside the tables were generated based on the prior results due to the inaccessibility of the robot arm for that position.

**Figure 12:**
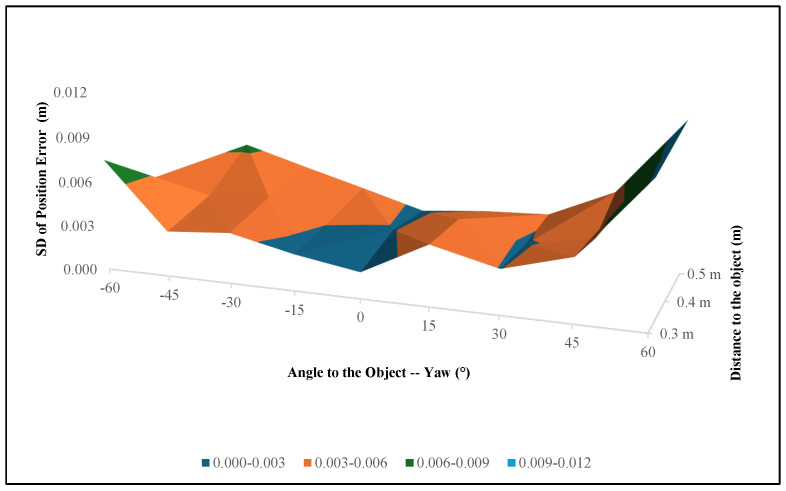
SD surface plot for the globe valve given by Table 4. A blue-colored surface in the plot indicates the accepted threshold range.

From the data in Table 2 and Table 3, it is clear that the position error generally increases as the yaw angle deviates further from 0°. The smallest errors occur when the angle is closer to 0° and 15°, particularly at distances of 0.3 m and 0.4 m away from the object. Specifically, the error is lowest at 0° and ±15° with distances of 0.3 m and 0.4 m. For example, at a distance of 0.3 m, the position error at 0° is 0.00537 m, which is an improvement compared to the previous research by Faria et al., where the error was approximately 0.007 m [42]. However, as the distance from the object increases to 0.5 m, the position error also increases, especially at more extreme angles such as −60° and 60°. At these angles, the errors are significantly higher, reaching up to 0.04712 m at 60° for a 0.3 m distance. The surface plot in Figure 11 further illustrates this trend, showing a clear increase in position error at the more extreme angles and longer distances. This plot reveals that the system’s accuracy is highest near the central region (close to 0°) and diminishes as the angle and distance increase. The standard deviation (SD) values for position error also increase as the yaw angle deviates further from 0°, as shown in Figure 12.

The orientation error results present a more complex picture compared to the position errors, indicating that the system’s accuracy is less predictable and more sensitive to variations in angle and distance. The orientation error is measured in degrees, and an accepted threshold of 10° was set for this evaluation. At a distance of 0.3 m, the orientation error generally remains below the 10° threshold across most angles, except at extreme angles such as 60° (18.367°). This finding suggests that the system performs better when the object is closer to the camera. Unlike position error, however, the orientation error does not have any notable error distribution pattern, as shown in the surface plot in Figure 11.

#### 7.2.2. Pose Estimation Results for Ball Valve

**Figure 13:**
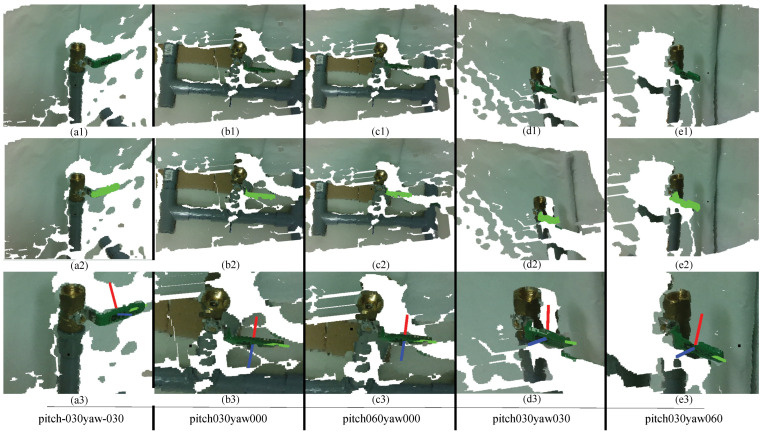
Experimental results for the 3D object recognition (sub-figures a2–e2) and pose estimation (sub-figures a3–e3) of the ball valve from different yaw and pitch axes.

The ball valve was not symmetric; therefore, we also collected data from different pitch and yaw axes. Some examples of different pitch and yaw axes for the ball valve are shown in Figure 13. In Figure 13(a1–e1), the scene is provided, in a2–e2, the object recognition is presented, and in a3–e3, the pose estimation for different pitch and yaw angles is presented.

**Figure 14:**
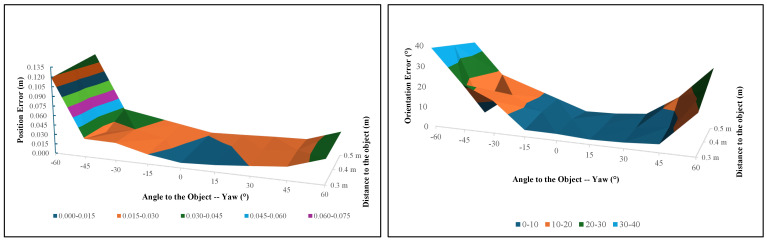
Surface plot for Table 5 and Table 6 for the ball valve. The left plot represents position error, while the right plot represents the orientation error at different yaw angles and distances from the object. A blue-colored surface in the plot indicates the accepted threshold range.

Table 5 and Table 6 and Figure 14 show how the position error changes according to the different yaw angles and different distances from the ball valve. The bold and italic values inside the tables were generated based on the prior results due to the inaccessibility of the robot arm for that position, while the yellow highlighted positions cannot be recognized properly due to the angle of the object.

The data in Table 5 and Table 6 reveal that the position error generally increases as the distance from the object increases, particularly at more extreme yaw angles. At a closer distance of 0.3 m, the system maintains a position error below 3 cm (0.03 m) across most angles, with the smallest error recorded at 0°, measuring 0.00784 m. At the more extreme angles of −60° and 60°, however, the errors increase significantly, with values of 0.118745 m and 0.037245 m, respectively, both of which exceed the 1.5 cm threshold. When the angles become negative, the errors increase compared to positive angles because the handle of the ball valve is less visible to the camera. Hence, it is harder to recognize the ball valve at negative yaw angles. At a distance of 0.4 m, the system continues to perform adequately within the central angle range, maintaining errors within the acceptable range. The lowest error at this distance is 0.00784 m at 0°. However, similar to the closer distance, the errors increase notably at higher degrees. When the distance from the object increases to 0.5 m, the position error becomes more pronounced across all angles. The position error results for the ball valve indicate that the proposed system performs adequately within an acceptable threshold at closer distances and moderate angles. Specifically, at a distance of 0.3 m, the system consistently achieves position errors below 1.5 cm (0.015 m) for ±15°, which is within the acceptable range for precise robotic manipulation.

At a distance of 0.3 m, the orientation error remains within the acceptable range for most angles, with errors below 10° at angles like −15° (3.333°) and 0° (3.199°). However, the errors increase substantially at the extreme angles of −60° and 60°, where they reach 38.83° and 19.55°, which are well beyond the acceptable threshold. At 0.5 m, the system performance shows a similar trend. While the orientation error is 0° (2.303°), it again exceeds the acceptable range at extreme angles, with errors of 18.026° at −45° and 14.269° at 45°. The errors at these extreme angles indicate that the system struggles to maintain accurate orientation estimation at greater distances. Unlike position error, orientation error distribution fluctuates at some yaw angles, specifically 0.4 m away at −45°. It is clear that when it comes to negative yaw angles, the orientation and position errors are higher than those of positive yaw angles. The reason behind this finding is that at negative yaw angles, the valve in the ball valve is not visible and is blocked by the body of the ball valve.

**Figure 15:**
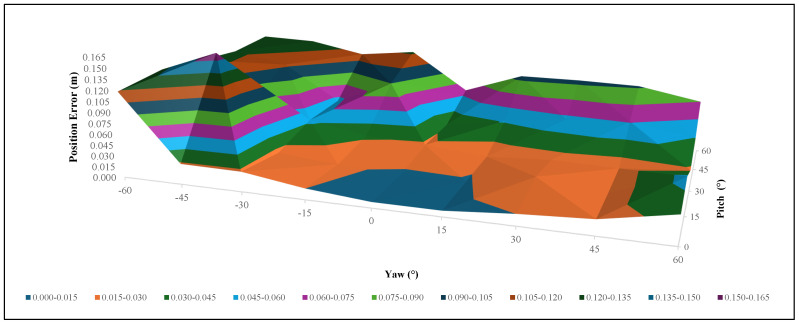
Surface plot for Table 7 showing the position errors of the ball valve results at different pitch and yaw angles. A blue-colored surface indicates the accepted threshold range.

From the above results, it is clear that when the distance from the object is 0.3 m, the position and orientation errors are minimal. The analysis of the ball valve was performed for different pitch and yaw angles at a 0.3 m distance, as shown in Table 7 and Figure 15. The data show that the position error remains below this threshold, primarily at lower pitch angles and moderate yaw angles. For example, at a pitch angle of 0° and a yaw angle of 0°, the position error is minimal, at 0.00784 m. However, as the pitch and yaw angles increase, the position errors also tend to increase, often exceeding the acceptable threshold. The surface plot further illustrates the system’s performance across the tested angles, indicating that the system is most reliable at lower pitch and yaw angles.

### 7.3. Determining the Green and Yellow Zones

To determine the green and yellow zones, the results in Figure 11, Figure 14 and Figure 15 were considered. It is clear that the ±15° area from the front of the object (pitch000yaw000) produces minimal error values regarding both position and orientation, being is lower than the threshold values given. The ±15° area was considered as the green zone, and the rest of the area (from ±15° up to ±60°) was considered as the yellow zone. As in Figure 2, if the robot arm is in the yellow zone, it will first move to the green zone and again capture the PC and then manipulate the valve.

### 7.4. Dexterous Manipulation Results

**Figure 16:**
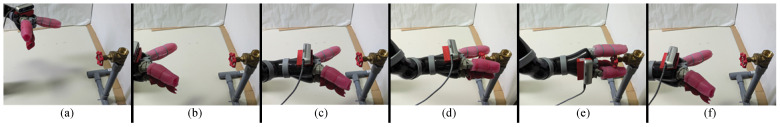
Steps for globe valve manipulation. (**a**) starting point, (**b**) moving to the green zone, (**c**) standby position, (**d**,**e**) manipulation of the valves, and (**f**) final position.

**Figure 17:**
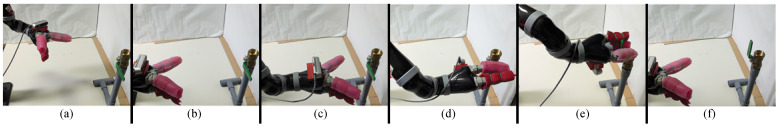
Steps for ball valve manipulation. (**a**) starting point, (**b**) moving to the green zone, (**c**) standby position, (**d**,**e**) manipulation of the valves, and (**f**) final position.

After determining the green zone and yellow zone, the dexterous manipulation module was tested. Figure 16 and Figure 17 show examples of the manipulation of globe and ball valves. In these figures, a, b represent the valve moving from the yellow to the green zone, c shows the arm moving to the standby position, d, e illustrate the manipulation of the valves, and finally f illustrates the final position of the robot arm.

## 8. Conclusions

In this research, we developed and evaluated a system for object recognition, pose estimation, and dexterous manipulation, focusing on industrial valves using a JACO robotic arm equipped with an Intel RealSense D435 camera. The proposed system was implemented using the PCL in C++, and experiments were conducted under controlled conditions to assess its performance across various scenarios. This research is particularly well suited for real-world environments, such as factories, where lighting conditions are controlled and consistent, ensuring that the chosen algorithms can perform reliably and maintain high accuracy in object recognition and pose estimation without the need for complex recalibration.

One of the key contributions of this research was the development of a novel, more accurate method for object recognition and pose estimation using a model-based approach. The object recognition module was a critical component of this system, comprising three submodules: scene segmentation & integration, geometrical primitives recognition, and model recognition. The color-based clustering and integration approach enhanced object detection by leveraging both geometric and color information, enabling the more accurate segmentation of PC data. A dynamic cluster merging algorithm was introduced to address the noise challenges in the real-time captured data, which ensured that the target objects, such as the valve handles and bodies, were effectively clustered. The RANSAC algorithm was employed to recognize geometric primitives within the segmented data, allowing for the accurate identification of valve components. This approach proved to be both robust and computationally efficient, supporting real-time or near-real-time applications. The target objects could be recognized well in this methodology in the given range of testing (up to ±60°).

Another major contribution was the introduction of a new technique to increase pose estimation accuracy by adjusting the camera position. Position and orientation errors were extensively analyzed for both globe and ball valves under different yaw and pitch angles. The results indicate that the system performs reliably within the acceptable error thresholds when the object is positioned within ±15° of the camera’s direct view (the green zone). In this zone, both position and orientation errors were minimal, confirming the system’s capability to estimate the pose and manipulate the valves accurately. However, as the angles became more extreme (up to ±60°) and the distance from the object increased, the errors escalated, particularly in the case of orientation estimation for the ball valve. These areas were classified as the yellow zone, where accuracy was diminished, and further refinement was necessary.

Finally, the research also aimed at the creation of a robot motion model for dexterous manipulation, specifically to open and close various valves efficiently. This was realized by dividing the manipulation strategy into two parts: direct execution in the green zone and camera repositioning in the yellow zone. By moving the camera to optimal angles when accuracy was compromised, the system was able to execute manipulation tasks more reliably, addressing the challenges posed by complex valve geometries and orientations. Our research findings demonstrate that while the proposed system is effective under specific conditions, its accuracy can be significantly affected by the orientation and distance of the object relative to the camera.

## 9. Future Works

The current system has been tested primarily on globe and ball valves, but the industrial landscape includes a diverse range of valve types with varying geometrical features. A significant area for future work involves applying this algorithm to a broader spectrum of valves. Since the system utilizes a JSON file to store geometrical models, adding new valves should be straightforward. This JSON-based approach allows for the easy extension of the system’s capabilities, enabling the integration of new valve types without the need for extensive reconfiguration. By expanding the database of geometric models and testing the algorithm across a wider array of valves, the system can be more versatile and applicable to a broader range of industrial applications.

While the current object recognition system has shown effectiveness in controlled environments, some applications often involve challenging conditions where noise and environmental factors can significantly impact performance. Future work should focus on improving the robustness of the object recognition system to manage noisy PC data more effectively. Enhancements include developing advanced noise reduction techniques and incorporating machine learning algorithms that are resilient to variations in lighting, reflections, and other environmental disturbances. By improving the system’s ability to operate under noisy conditions, particularly at more extreme angles and greater distances, the overall reliability and accuracy of the system can be significantly enhanced. This will be critical for deploying the system in real-world industrial settings, where environmental control is often limited.

## Figures and Tables

**Table 1:** Summary of previous research for object recognition, pose estimation, and dexterous manipulation.

Research	Objective	OR	PE	DM
Tsai et al. [1]	Developed a system for simultaneous object recognition and pose estimation using RGB-D images.	yes	yes	no
Guo et al. [22]	Developed a method for object detection and 6D pose estimation in complex point clouds.	yes	yes	no
Parisotto et al. [15]	Used a multi-view convolutional network for simultaneous object recognition and pose estimation.	yes	yes	no
Jantos et al. [23]	Presented a transformer-based framework for multi-object 6D pose estimation from single RGB images.	yes	yes	no
Tu et al. [28]	Created a multi-modal fusion system for object-in-hand pose estimation using vision and tactile data.	yes	yes	yes
Park et al. [29]	Automated the process of annotating 6D object poses during dexterous manipulation using robot kinematics.	no	yes	yes

Note: OR: object recognition, PE: pose estimation, DM: dexterous manipulation.

**Table 2:** Mean position error (m) at different yaw angles and distances from the object.

Distance	Angle in Relation to the Object (°)								
	−60	−45	−30	−15	0	15	30	45	60
**0.3 m**	0.02534	0.01961	0.01439	0.01010	0.00537	0.00923	0.01046	0.01879	0.04712
**0.4 m**	0.03845	0.02804	***0.02230***	***0.01480***	0.00820	0.00931	0.01082	0.02521	***0.05110***
**0.5 m**	0.04452	0.03785	***0.02920***	***0.02050***	0.01183	0.01041	0.01098	0.02670	***0.05500***

Note: We could not measure the bold and italic values due to the inaccessibility of the robot arm for that position.

**Table 3:** Orientation error (°) at different yaw angles and distances from the object.

Distance	Angle in Relation to the Object (°)								
	−60	−45	−30	−15	0	15	30	45	60
**0.3 m**	3.212	2.359	10.546	10.892	3.293	5.153	8.082	5.767	18.367
**0.4 m**	5.544	3.626	***9.600***	***9.171***	5.953	7.659	10.169	20.113	***18.359***
**0.5 m**	9.068	9.082	***8.395***	***7.709***	7.022	5.483	10.601	5.767	***18.355***

Note: We could not measure the bold and italic values due to the inaccessibility of the robot arm for that position.

**Table 4:** Mean position error (m) for SD at different yaw angles and distances from the object.

Distance	Angle in Relation to the Object (°)								
	−60	−45	−30	−15	0	15	30	45	60
**0.3 m**	0.00751	0.00306	0.00341	0.00245	0.00175	0.00402	0.00295	0.00419	0.00938
**0.4 m**	0.00484	0.00404	***0.00430***	***0.00310***	0.00289	0.004048	0.00283	0.00367	***0.00990***
**0.5 m**	0.00286	0.00635	***0.00510***	***0.00390***	0.00265	0.003046	0.00323	0.00520	***0.01040***

Note: We could not measure the bold and italic values due to the inaccessibility of the robot arm for that position.

**Table 5:** Position error (m) at different yaw angles and distances from the object.

Distance	Angle in Relation to the Object (°)								
	−60	−45	−30	−15	0	15	30	45	60
**0.3 m**	0.11875	0.02816	0.02721	0.01505	0.00784	0.01044	0.01512	0.02013	0.03725
**0.4 m**	*0.12590*	0.02016	***0.03000***	***0.02030***	0.01166	0.01449	0.01830	0.02346	***0.03760***
**0.5 m**	*0.13310*	0.0425	***0.03370***	***0.02480***	0.01597	0.01756	0.01948	0.02082	***0.03790***

Note: We could not measure the bold and italic values due to the inaccessibility of the robot arm for that position, and italic positions cannot be measured due to object recognition.

**Table 6:** Position error (°) at different yaw angles and distances from the object.

Distance	Angle in Relation to the Object (°)								
	−60	−45	−30	−15	0	15	30	45	60
**0.3 m**	38.83	22.468	15.827	3.333	3.199	2.343	2.785	3.890	19.550
**0.4 m**	*36.613*	2.717	***13.399***	***6.345***	4.274	1.479	3.013	5.433	***24.740***
**0.5 m**	*34.396*	18.026	***12.785***	***7.544***	2.303	2.689	4.180	14.269	***29.930***

Note: We could not measure the bold and italic values due to the inaccessibility of the robot arm for that position, and italic positions cannot be measured due to object recognition.

**Table 7:** Pitch vs. yaw measurement values when the distance from the object is 30 cm.

Pitch (°)	Yaw (°)								
	−60	−45	−30	−15	0	15	30	45	60
**0**	0.11875	0.02816	0.02721	0.01505	0.00784	0.01044	0.01512	0.02013	0.03725
**15**	0.13024	0.15943	0.02473	0.01758	0.01217	0.01353	0.02989	0.01961	0.05002
**30**	0.12648	0.07845	0.04799	0.03220	0.02709	0.01894	0.02345	0.02101	0.02797
**45**	0.12420	0.10788	0.05550	0.08996	0.03235	0.04648	***0.05640***	***0.06110***	***0.04440***
**60**	***0.13360***	***0.13030***	0.11347	0.12243	0.06547	0.09477	0.09433	***0.09180***	***0.07500***

Note: We could not measure the bold and italic values due to the inaccessibility of the robot arm for that position.

## Data Availability

The data can be found online at https://github.com/udaka21/Object_Rec_Pose_Est (accessed on 14 September 2024).

## Abbreviations

The following abbreviations are used in this manuscript:

PC	Point cloud
RANSAC	Random sample consensus
IK	Inverse kinematics
6-DOF	Six degrees of freedom
CAD	Computer-aided design
JSON	JavaScript Object Notation
PCL	Point cloud library
SD	Standard deviation
